# Scoping Review of Systems to Train Psychomotor Skills in Hearing Impaired Children

**DOI:** 10.3390/s18082546

**Published:** 2018-08-03

**Authors:** Victor M. Peñeñory, Cristina Manresa-Yee, Inmaculada Riquelme, Cesar A. Collazos, Habib M. Fardoun

**Affiliations:** 1 Multimedia Engineering Program, University of San Buenaventura, Ave. 10 de Mayo, La Umbria, 760031 Cali, Colombia; 2 Department of Mathematics and Computer Science, University of the Balearic Islands, Crta. Valldemossa km. 7.5, 07122 Palma, Spain; 3 Department of Nursing and Physiotherapy, Institute of Health Sciences Research, University of Balearic Islands, 07122 Palma, Spain; inma.riquelme@uib.es; 4 Systems Program, University of Cauca, Cl. 5 No. 4–70, 190001 Popayan, Colombia, ccollazo@unicauca.edu.co; 5 King Abdulaziz University, Al Ehtifalat St, Al-Ruwais, 21589 Jeddah, Saudi Arabia; 6 Teaching Excellence Department, Ahlia University, Bld 41 Rd 18, Al Hoora 310, Bahrain; hfardonn@ahlia.edu.bh

**Keywords:** psychomotor development, psychomotor deficits, hearing impairment, children with special needs, sensors, interactive systems

## Abstract

Objectives: The aim of this work is to provide a scoping review to compile and classify the systems helping train and enhance psychomotor skills in hearing impaired (HI) children. Methods: Based on an exhaustive review on psychomotor deficits in HI children, the procedure used to carry out a scoping review was: select keywords and identify synonyms, select databases and prepare the queries using keywords, analyze the quality of the works found using the PEDro Scale, classify the works based on psychomotor competences, analyze the interactive systems (e.g., sensors), and the achieved results. Results: Thirteen works were found. These works used a variety of sensors and input devices such as cameras, contact sensors, touch screens, mouse and keyboard, tangible objects, haptic and virtual reality (VR) devices. Conclusions: From the research it was possible to contextualize the deficits and psychomotor problems of HI children that prevent their normal development. Additionally, from the analysis of different proposals of interactive systems addressed to this population, it was possible to establish the current state of the use of different technologies and how they contribute to psychomotor rehabilitation.

## 1. Introduction

Hearing loss in children can vary from mild to profound [1]. Hearing damage can occur due to diseases, infections or vestibular damage producing hearing loss. This problem can interfere with the sensorimotor function, causing delays for hearing impaired (HI) children, in their psychomotor development, i.e., motor skill, balance, dynamic coordination, visual-motor coordination performance, among other aspects [2,3,4,5].

Psychomotricity integrates the cognitive, emotional, symbolical and physical interactions in the individual’s capacity to be and to act in a psychosocial context. Motor development lays the basis for higher complex psychological abilities, such as emotion regulation or symbolism. Thus, the adequate acquisition of basic psychomotor areas, such as body schema (an essential part of body awareness, body image and self-esteem), gross motor skills (i.e., posture, balance), fine motor skills, space, time and rhythm are determinant for the development of cognition, emotion and social interactions.

As hearing impairment may impact the psychomotor development of HI children, the objective of the present work was to revise the literature to compile and classify the systems used to help train psychomotor skills in HI children. Particularly, the aim of this review was to compile the endeavors being done in the field, to show which areas could be enriched with the use of technologies and interactive systems. Technology is being increasingly incorporated to rehabilitation processes, providing additional benefits such as providing real-time feedback or making the exercises more attractive, increasing adherence to treatment [6,7].

## 2. Background

### 2.1. Psychomotor Limitations in Hearing Impaired Children

Psychomotor abilities are usually classified into fundamental motor skills (i.e., body schema, body image, posture, balance and coordination), perceptual motor skills (i.e., space, time and rhythm) and cognitive skills (executive functions, such as memory or reasoning processes). The present study is focused on psychomotor abilities associated with motor skills, in relation with psychological processes.

#### 2.1.1. Fundamental Motor Skills

HI children typically have lower scores on psychomotor scales than normal hearing (NH) children [2,8]. For instance, more than the 30% of HI children showed retardation in the acquisition of head control or independent gait [9]. Poor motor performance does not appear to affect self-efficacy in HI children [10], but it has been related to language deficiencies, poorer symbolic play, emotion dysregulation and social difficulties in interacting with other children [8,11]. Children with cochlear implanta show a drop in their gross motor performance coinciding with surgery, and a period of at least two years is needed to recover the developmental delay [12]. HI children exhibit worse gait performance than NH children, with abnormal ground reaction forces, higher propulsion and lower free movements [13,14]. Higher hearing impairment determines worse postural recovery and gait performance [14,15] and hearing aids and cochlear implants may help promote improvements in gait and stability during walking [16].

Body perception. Hearing impairment disturbs the experience of the own body and body-related abilities. Body perception deficits may play an important role in the action performance and be the cause of many unexplained daily difficulties suffered by HI children [17].

Posture is determined by the internal representation of the body in the surrounding space. Posture is permanently adjusted to environmental modifications by the continuous central integration of multisensory inputs that trigger motor commands for allowing stability. Thus, the unceasing inputs coming from the visual, vestibular and proprioceptive systems provide the brain with information of spatial context, head movement and position, and movement and position of the different body segments, respectively, which is crucial for posture maintenance and balance. HI children show higher postural instability and less ample head movements than NH children, which may indicate damage of the vestibular system [3,18]. Postural stability of HI individuals improves with adaptive sensory compensation (visual and vestibular) [19]; thus, HI children may benefit from exercise programs aimed at improving the body posture maintenance and balance control [20].

Balance is the ability to adapt postural control to be stable on different modifications of the environment. Balance develops during childhood, becoming a paramount parameter for the achievement of gross motor skills, such as running or jumping/standing on one leg [21]. Auditory inputs provide additional cues to control balance, creating a hearing “map” of surroundings that NH individuals use to maintain balance control and reduce postural sway [16,22]. HI children may experience balance difficulties, especially those with vestibular deficits [23,24] or within the first year of cochlear implants, when children exhibit higher rates of vestibular loss [25,26,27,28]. Thus, HI children have shown lower stability limits, faster and higher body sway, and higher energy expenditure to keep balance than NH children, indicating a deficit in static and dynamic balance [14,26,29,30]. HI children tend to use visual feedback in a higher amount than NH children, especially when balance is compromised by sensory disturbance (e.g., irregular surface) and the risk of falling increases [29,30]. Hearing aids, vestibular rehabilitation and physical exercise have proven effective to enhance vestibular adaptation and improve balance in HI children or after cochlear implant surgery [22,25,31]. HI individuals with additional vestibular deficits seem to exploit auditory cues in a higher degree, due to the reduced sensory redundancy [22]. On the other hand, vestibular dysfunction and its resulting balance deficits have been identified as risk factors for cochlear implant failures [32].

Coordination is defined as the global functionality of muscle groups, in a specific temporal order, resulting in the progressive contraction of agonists and the simultaneous inhibition of antagonists to achieve a motor outcome. Coordination is present in all motor functions (e.g., visuomotor, bimanual …). In gross motor function, coordination is refined later in HI children, who will achieve accurate execution of large body actions (e.g., running) in older ages than NH children [12]. Thus, motor skills such as catching a ball, requiring visuomotor, spatial and temporal coordination, are impaired longer in HI children, with higher reaction times than NH children [33]. Auditory deprivation also affects motion perception [34] and motor sequence learning [35]. Fine motor function (i.e., manipulation or manual dexterity) experiences a delay when prelingually HI children grow up [35,36]. Associations between fine motor function and receptive and expressive language in HI children post-implant [36] suggest common brain networks and seem to indicate that auditory deprivation leads to atypical motor and language development. Visuomotor integration development is impacted by early auditory and linguistic experience and seem to elicit different cognitive resources in HI children [36].

#### 2.1.2. Perceptual Motor Skills

Spatial skills are defined as the mechanisms allowing the awareness of object position and its relationships in the environment [37]. HI children compensate auditory deprivation highlighting attention to stimuli in the near and far space visual fields (to visual central stimuli in far space and to peripheral visual stimuli in near space) [38,39]. Other adaptation mechanisms in HI children are higher location memory [40] and higher visual and tactile orientation in the allocentric frame of reference (encoding the position of an object in relation to others), which allows a fast attention to targets [38,41]. Nevertheless, the discrimination at midline and lateral positions [29] and the egocentric frame of reference (encoding the position of an object in relation to the own body) seem to be abnormal in HI children. In this sense, goal-directed movements towards the objects, based in the egocentric frame of reference, are slower than in NH [41]. In addition, auditory deprivation affects brain spatial organization; thus, in contrast with NH children, who have shown right hemisphere activation for spatial attention, an atypical bilateral or left hemisphere activation has been seen in HI children [40,42]. Furthermore, spatial cognition is promoted by an expertise in spatial language [43] and language absence results in poor performance on non-linguistic spatial tasks, particularly those combining different spatial representations [44]. For example, a consistent linguistic marking of left-right is associated with search under disorientation or a consistent marking of ground information is associated to search in rotated arrays in deaf signers [43]. In contrast with temporal abilities, spatial competence are likely to improve in HI children, particularly with the use of sign language [45].

Temporal skills recall the order, timing and sequence of stimuli [46]. Hearing loss diminishes the capacity of using fine temporal signals for recognizing speech and non-speech cues out of the variable environmental noise [47]. Temporal processing of proprioceptive and tactile signals is also compromised in children with HI [48]. Event-related brain potentials revealed less precise phonological representations of rhythm of oral language or location of sign language in HI, compared to NH children [49]. Nevertheless, cochlear implant users had similar performance in temporal organization tasks than NH individuals [50]. On the other hand, cross-modal plasticity driven by experience may allow HI individuals a normal performance when synchronizing temporally discrete visual stimuli and visual timing [51].

## 3. Methods

### 3.1. Search Strategy

The following databases were used for the search: Science Direct, ACM Digital Library, IEEEXplore, NCI (PubMed, Bethesda, MD, USA), Springer Link and CiteSeerX. The following key words were used: auditory deficiencies, children, psychomotor, Human Computer Interaction (HCI), interactive systems, serious games and synonyms (e.g., hearing impairments, videogames, virtual systems). During the bibliographic analysis, we identified different contributions to the area of psychomotor training in HI children. Inclusion criteria were: studies whose title or abstract was related with helping train and enhance psychomotor skills in hearing impaired (HI) children, studies published in the last ten years in recognized journals or international events in the areas of health, biomedicine, computer science and HCI and studies describing the electronics or interactive elements used in systems aiming at the psychomotor skills improvement. Exclusion criteria were: studies addressing cognitive psychomotor skills and repeated studies. Thirty eight papers were identified in the first search (applying the key words), which were reduced to 13 after applying the inclusion and exclusion criteria. These contributions were classified into fundamental motor skills (posture, coordination and balance) and perceptual motor skills (spatial, temporal and rhythm). No references related to body image or body schema were found.

### 3.2. Quality

The PEDro Scale was used to evaluate the quality of the 13 papers found in the search and therefore, included in the review. This scale, based on the consensus of health experts, aims at identifying the works that have sufficient validity and statistical information to make their results interpretable [52]. The PEDro Scale evaluates the quality using eleven criteria, such as the selection criteria, the random assignment of the study subjects, the participation of the therapists and evaluators, or the comparison and visualization of the data. The criteria of the PEDro Scale assigns three values of quality: low risk of bias (1), unclear risk of bias (2), high risk of bias (3).

**Figure 1:**
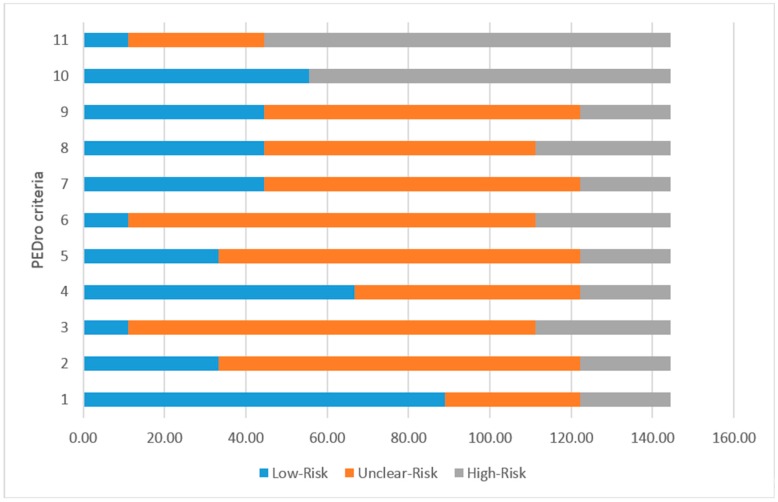
Results of the total scores of the PEDro Scale for the works included in the present review.

In the case of our review, papers following our inclusion criteria were scarce. The PEDro scale showed high values for unclear risk of bias (2) in most of the studies. In many of those studies, the evaluation with patients did not follow the standard procedures, which reduces the reliability of the data. One of the reasons explaining this fact, could be that most of the papers were published in conferences focused on human-computer interaction, therefore, the objective of the work was the presentation of the design and evaluation of the system, instead of analyzing a long-term progress of psychomotor skills. Figure 1 shows the quality of the works included in the review, according to the PEDro scale criteria.

### 3.3. Systems to Train Psychomotor Skills

#### 3.3.1. Fundamental Motor Skills

In this section, we present the interactive systems and applications that aimed at training the fundamental motor skills of HI children, which involved coordination, posture and balance.

Iversen and Kortbek [53] built an interactive floor for children with cochlear implants to interact with body movements. Their proposal includes a set of games aiming at language training and implant calibration.

To rehabilitate the upper body limbs, Wille et al. [54] used a VR environment and tested the system with children during three weeks. The games presented in the environment, helped children to rehabilitate the motor skills without stress. Further, children improved their hand function.

Marnik et al. [23] developed a system that can be used for therapy and education based on natural body movements and gestures interaction. The system uses computer vision techniques to detect the body movement and presents an attractive application to engage the user. The system is addressed to children with developmental problems, e.g., ASD or HI children, and presents simple physical exercises or gives instructions, such as “standing on a specified place and rise hands”, for the child to follow. Egusa et al. [55] developed a system based on Microsoft Kinect to allow HI children to enjoy and participate in a puppet show, while developing their body expression.

Radovanovic [56] examined the influence of specialized software on the visual-motor integration of profoundly deaf children. The evaluation was done with 70 children, 43 of whom formed an experimental group that used the computer once a week for five months. Results showed higher scores for the experimental group in a subtest of the Acadia test, but they were significantly higher only for seven-year-olds. Nevertheless, the authors supported the benefits of using videogames to improve visual-motor skills.

Noorhidawati et al. [57] compiled situations in which children engage with mobile apps to better understand how they learn through such interaction. They carried out a qualitative approach by observing 18 pre-schoolers interactions when using 20 mobile apps. The analysis demonstrated that learning in this environment takes place through cognitive, psychomotor-based, and affective means. Further, the use of these apps can help in improving the aforementioned skills.

To improve the body posture when performing physical exercises such as squats, Conner et al. [58] proposed a system based on computer vision techniques (via Microsoft Kinect). Results of the evaluation showed an improvement of the children’s body posture when following the instructions of the system.

Zhu et al. [59] used tangible objects to interact with digital elements in a role-playing collaborative game. Children trained their body expression and movements while playing with their teammates by fighting against the enemies, waving weapons according to different rhythms.

#### 3.3.2. Perceptual Motor Skills

The rehabilitation of perceptual motor skills develops the perception of space, time and rhythm in HI children. However, few contributions were found by our review.

To train rhythm, Jouhtimäki et al. [60] developed an educational tool which aimed at improving the children’s skills to identify and produce rhythmic patterns. These abilities also support language perception and literacy.

Pérez-Arevalo et al. [39] designed a similar game as the previous work, but they selected to deploy it in mobile devices. The proposal presents a game to train rhythm and coordination (visual motor) for HI children based on visual and auditory stimuli. They evaluated the system with nine children who had cochlear implants or used auditive aids. Children enjoyed the game and they all liked the emotional aspects of the design such as the customization of the main character, the graphical design of the character and the app or the storyline.

Correa et al. [58] proposed the construction of an interactive multimodal system for rehabilitating skills such as hand-eye coordination, memory, rhythm and tempo. The proposal is a multimodal system, similar to a piano, that allows visualization and feedback of musical notes through vibrations and exploits the perceptual phenomenon of synesthesia, which relates colors and sounds. The goal of the system is the improvement of the rhythmic perceptual skills through coordination, visualization of colors, and tempo.

Sogono and Richards [61] proposed the design of a game involving electronic elements, which would allow HI children to perform sound localization activities in closed spaces. In addition to this, they also proposed templates to facilitate the design of this type of multisensory environments.

Finally, Aditya et al. [62] proposed a project to help children understand better abstract concepts such as time, from the use of tangible objects together with visual feedback. With the proposal they built an intelligent clock that represented each of the activities that the child carried out in his or her daily life.

#### 3.3.3. Summary of Works

Table 1 shows the classification of the works according to the type of skill that they impact (fundamental motor skills and perceptual motor skills).

## 4. Results

We analyzed the sensors used in the systems found in the research to study the current technology used to work psychomotor skills in HI children.

### 4.1. Computer Vision

When the computer is able to “see”, it can use the visual information for interaction purposes. The use of one or more cameras, i.e., webcams or Microsoft Kinect, allows the computer to sense the user and his/her actions, gestures or postures. Works by Egusa et al. [55] or Conner et al. [63] used RGBD cameras to sense the user’s body posture and the gestures performed, whereas, the work by Marnik et al. [23] focuses on computer vision algorithms to recognize gestures. During the assessment, researchers concluded that the use of computer vision techniques in motor training allowed participants to be immersed in the proposed activities and they found them motivating.

### 4.2. Interactive Floors

Interactive floors are frequently classified into sensor-based or vision-based. In the case of Iversen and Kortbek [53], they combine both types to construct an interactive floor setup with vision tracking limb contact points from below the floor surface. During the evaluation of their system, the authors found that interactive floor-based systems invited participants to interact actively and collaboratively, allowing an exchange of knowledge and communication between users. It also offered children a way to create their own games related to the exercises they had to do, which increased motivation and engagement.

### 4.3. Touch Screens

Touch screens are present in mobile devices, tablets and mobile phones. The increase of these devices and the familiarity with this interaction mode helps children to focus directly on the tasks to carry out [39,62]. Researchers used tablets [39] or smart watches [62] to teach children rhythm and eye-hand coordination. During the evaluation, authors stated that interactive systems based on game actions related to activities of daily living (ADL), and with a smooth learning curve, allowed the children to quickly develop skills related to the rehabilitation goals in an amusing and motivating way.

### 4.4. Mouse and Keyboard

Mouse and keyboard are still the traditional input devices to desktop computers and have been used to control the interaction of applications for HI children [60]. During the evaluation of this proposal, authors found that using these devices in an interactive game supported children with some kind of disability to acquire rhythm patterns, which may enhance language development.

### 4.5. Tangible Artifacts

By using Arduino or similar hardware boards, researchers created artifacts for HI children to hold and manipulate, which controlled the interaction and informed about their position or movement through accelerometers [59]. In the evaluation of the system, participants were more motivated to participate collaboratively in the activity due to the use of tangible elements such as swords, shields and wands. However, some participants had difficulties due to the lack of tempo management and music knowledge, therefore, authors concluded that visual aids should be used.

### 4.6. Multimodal Systems

We also found sensors integrated in multimodal systems, which combined different input and output modalities, such as touch, computer vision and tangible objects together with visual and haptic feedback [58,61]. Researchers concluded that the use of non-traditional interaction devices made participants consider the activities to be entertaining and attractive because they invited to use different senses and effectors.

### 4.7. VR Devices

The use of VR devices such as data gloves and head-mounted displays (HMD) have also been used [54] to interact in a VR environment. During the evaluation [54], some participants presented evidence of improvement in activities proposed by the interactive system. However, despite the fact that participants showed a high degree of motivation when performing the different actions in the game, some did not progress as expected, showing a low level of progress in therapy.

## 5. Discussion

### 5.1. Computer Vision

The results obtained by [23,55,63] were achieved thanks to the use of technologies and sensors that allowed the tracking of the different movements of the patient’s body, offering an accurate feedback in real time of their movements and informing them of the correctness. Additionally, when presenting these exercises as play activities, participants felt more comfortable and motivated with the activity, because they were immersed in the exercise [64,65].

### 5.2. Interactive Floors

The use of a contact surface, together with a projection and a camera, allowed the creation of different interactive spaces. Users had fun while performing physical activities [66]. Some of the results of the use of this technology can be observed in [53] where, based on the proposed interactive environment, children were more motivated to carry out the rehabilitation activities and the technology provided allowed them to be in a playful and collaborative environment with the freedom to make different activities.

### 5.3. Touch Screens

The use of touch technologies allowed patients to use their upper limbs to train fine motor skills and hand-eye coordination [67]. The use of this type of technology in the proposals [39,62] allowed participants to visualize and interact with the applications in real time while training fine motor and hand-eye coordination skills.

### 5.4. Mouse and Keyboard

The acquisition of motor skills in upper limbs can be carried out with traditional devices such as keyboards and mice. The game to train rhythm skills proposed by [60], allowed the user to perform each of the activities comfortably following the patterns and exercises offered by the game.

### 5.5. Tangible Artifacts

Tangible objects can be used in motor training and play activities because they count with different sensors such as accelerometers, gyroscopes and radio frequency identifiers, which allow patients to interact directly with them. Further, the system outputs in real-time video and audio accordingly to the patient’s actions on the objects. The proposal by [59] clearly shows that the use of tangible objects contributed to a greater willingness to participate in the activities and collaborate with other children while performing the exercises and having fun.

### 5.6. Multimodal Systems

Multimodal systems allow the construction of interactive environments in which users can use different senses and carry out play activities that contribute to their psychomotor development [68,69,70,71]. This case can be clearly seen in [58,61] where the created devices involved different feedback, such as haptic and visual cues, that allowed the patient to perceive the information through multiple senses and react to the stimuli, causing the activity to be more engaging.

### 5.7. VR Devices

VR systems allow interact in a virtual world. Users can perform actions and receive a direct feedback through the objects of the virtual world with which they interact. This type of technology allows children with different types of psychomotor problems to carry out actions that cannot be done naturally in the real world, obtaining a satisfaction feeling while doing the activity. This fact is clearly observed in proposal [51], as the use of VR devices such as the glasses, helps children to participate in the rehabilitation process with a high level of motivation.

### 5.8. Limitations

This study has some limitations that must be taken into account for the correct interpretation of the results. The quality of the selected works, by the use of the PEDro scale, was performed only by one researcher; although PEDro criteria are clear and well-defined, the separate evaluation by different researchers would have allowed increasing the reliability of the scores.

## 6. Conclusions

HI children present deficits in their psychomotor development, which affect physical, social or emotional dimensions of their development. Therefore, research aiming at the improvement of the psychomotor skills of this population is needed. Based on an exhaustive review on psychomotor deficits in HI children, the aim of this work was to compile the endeavors being done in the field, to show which areas could be enriched with the use of technologies and interactive systems.

During the review it was evidenced the use of different interaction technologies such as touch screens, tangible objects, traditional devices (mouse and keyboard), cameras and VR devices that together with different electronic elements like Arduino, RFID or buttons, allowed the creation of interactive environments and aimed to create more entertaining and motivating activities.

The results of the analyzed proposals showed that HI children felt more satisfied with the activities. This feeling was due to the fact that the different technologies offered the children a real-time direct feedback on the performance of the different actions, which allowed them to feel part of their rehabilitation process. Thus, technology could increase the enjoyment of the rehabilitation sessions and increase the adherence to treatment, which is of paramount importance at these ages.

Despite these results, this review showed that there is a lack of tools to support HI children during their therapy, due to reasons such as the cost of the technology or the limitation to access children with this kind of disability. This should encourage the scientific and academic community to actively participate in the generation of interactive systems that address the psychomotor development needs of HI children in a motivating and attractive context, offering interactive tools that use low-cost digital and electronic elements. In addition, studies showed a high prevalence of unknown risk of bias, which made difficult to test their success in improving psychomotor skills in HI children. Many reasons can cause this low quality: lack of long clinical trials, cost of certain technologies and creation of systems, etc. Investigating the barriers in the use of the technology in clinical settings would help identify the essential reasons. Moreover, long-term studies investigating the effects in the improvement of psychomotor skills would increase the quality of the present knowledge.

## Figures and Tables

**Table 1:** Classification of contributions according to their impact on fundamental motor skills and perceptual motor skills.

Skills	Competences	Works
Fundamental motor skills	Coordination	[56]
	Balance	[23,53,54,55,57,59]
	Posture	[63]
Perceptual motor skills	Space	[61]
	Time	[62]
	Rhythm	[39,58,59,60]
